# Molecular Signatures of Cardiac Defects in Down Syndrome Lymphoblastoid Cell Lines Suggest Altered Ciliome and Hedgehog Pathways

**DOI:** 10.1371/journal.pone.0041616

**Published:** 2012-08-09

**Authors:** Clémentine Ripoll, Isabelle Rivals, Emilie Ait Yahya-Graison, Luce Dauphinot, Evelyne Paly, Clothilde Mircher, Aimé Ravel, Yann Grattau, Henri Bléhaut, André Mégarbane, Guy Dembour, Bénédicte de Fréminville, Renaud Touraine, Nicole Créau, Marie Claude Potier, Jean Maurice Delabar

**Affiliations:** 1 Univ Paris Diderot, Sorbonne Paris Cité, Unité de Biologie Fonctionnelle et Adaptative, EAC4413 CNRS, Paris, France; 2 Equipe de Statistique Appliquée, ESPCI ParisTech, Paris, France; 3 CRICM, CNRS UMR7225, INSERM UMR975, UPMC Hôpital de la Pitié-Salpêtrière, Paris, France; 4 Institut Médical Jérôme Lejeune et Fondation Jérome Lejeune, Paris, France; 5 Unité de Génétique Médicale, Faculté de Médecine, Université Saint-Joseph, Beirut, Lebanon; 6 Cardiologie pédiatrique, Cliniques Universitaires St Luc, Bruxelles, Belgique; 7 Service de Génétique, Centre Hospitalier Universitaire de Saint-Etienne, Saint-Etienne, France; Institut Jacques Monod, France

## Abstract

Forty percent of people with Down syndrome exhibit heart defects, most often an atrioventricular septal defect (AVSD) and less frequently a ventricular septal defect (VSD) or atrial septal defect (ASD). Lymphoblastoid cell lines (LCLs) were established from lymphocytes of individuals with trisomy 21, the chromosomal abnormality causing Down syndrome. Gene expression profiles generated from DNA microarrays of LCLs from individuals without heart defects (CHD^−^; n = 22) were compared with those of LCLs from patients with cardiac malformations (CHD**^+^**; n = 21). After quantile normalization, principal component analysis revealed that AVSD carriers could be distinguished from a combined group of ASD or VSD (ASD+VSD) carriers. From 9,758 expressed genes, we identified 889 and 1,016 genes differentially expressed between CHD^−^ and AVSD and CHD**^−^** and ASD+VSD, respectively, with only 119 genes in common. A specific chromosomal enrichment was found in each group of affected genes. Among the differentially expressed genes, more than 65% are expressed in human or mouse fetal heart tissues (GEO dataset). Additional LCLs from new groups of AVSD and ASD+VSD patients were analyzed by quantitative PCR; observed expression ratios were similar to microarray results. Analysis of GO categories revealed enrichment of genes from pathways regulating clathrin-mediated endocytosis in patients with AVSD and of genes involved in semaphorin-plexin-driven cardiogenesis and the formation of cytoplasmic microtubules in patients with ASD-VSD. A pathway-oriented search revealed enrichment in the ciliome for both groups and a specific enrichment in Hedgehog and Jak-stat pathways among ASD+VSD patients. These genes or related pathways are therefore potentially involved in normal cardiogenesis as well as in cardiac malformations observed in individuals with trisomy 21.

## Introduction

Trisomy 21 (Ts21) or Down syndrome (DS) is the most common human chromosomal aneuploidy at birth, and the only one with long-term viability. Congenital heart defects (CHD) are present in about 40–60% of newborns with DS [1]. Neonatal detection of cardiac defects followed by cardiac surgery has contributed to increased life expectancy of individuals with DS.

Cardiac abnormalities in DS include atrioventricular septal defect (AVSD) (with or without other CHDs), ventricular septal defect (VSD) (with or without other CHDs), isolated secundum atrial septal defect (ASD), isolated persistent patent ductus arteriosus (PDA), and isolated tetralogy of Fallot (TOF). The reported frequency of each defect varies between studies of different populations, indicating that genetic background or consanguinity may affect the CHD phenotype [2]–[8]. Additionally, analysis of Ts21 fetal hearts [9], showing that CHD phenotypes may include the small anomaly called linear insertion of atrioventricular valves (LIAVV), suggest that the number of individuals with Ts21 and LIAVV may be underestimated. Most of the cardiovascular abnormalities associated with Down syndrome can be detected during fetal development; however, percentages of cardiac defects detected in fetuses, mostly AVSD and VSD, vary between studies [10], [11]. Cardiac septal defects are typically observed during the first trimester and are frequently associated with an increase in nuchal translucency thickness.

The cellular and molecular events that control cardiac development are conserved among vertebrates. Heart development in humans occurs very early, from the third to eight weeks of gestation, beginning with a primitive tube that beats at 25 days gestation and ending in the four-chamber heart. Many steps occur after formation of the primitive heart tube, including looping, cell migration, cell transition, and septation events [12]–[14]. Many studies indicate that cardiac development is tightly regulated by a series of molecular signaling pathways and morphological events. However, many questions remain regarding the changes that occur to alter heart development in individuals with DS. For example, it is unclear to what extent secondary changes occur as a consequence of the aneuploid state, and which environmental factors combine with genetic causes to induce such changes.

Inter-individual differences in gene expression are likely to account for an important fraction of phenotypic differences, including susceptibility to common disorders. Recent studies have shown extensive variation in gene expression levels in humans and other organisms, and that a fraction of this variation is under genetic control [15].

Indeed, the genetic alterations in DS are not as simple as once believed: while genes on chromosome 21 (HSA21) are transmitted in 3 copies, all of those genes do not necessarily exhibit a straightforward 1.5-fold increase in expression. Gene expression may be regulated by dosage compensation such that only a subset of those genes exhibit the expected 50% increase in expression. A previous study using lymphoblastoid cell lines (LCLs) from diploid (2N) individuals and individuals with Ts21 showed that only 30% of HSA21 genes are significantly overexpressed [16]. For genes located on chromosomes other than 21, the effect of Ts21 could be relatively subtle or massively disruptive.

One hypothesis explaining the complicated genetics of DS proposes that gene expression changes on HSA21 are likely to affect the expression of genes on other chromosomes through the modulation of transcription factors, chromatin remodeling proteins, and related molecules or other targets. Thus, the dysregulation of pathways involved in heart development may cause the cardiac defects observed in DS. A second hypothesis implicating both environmental and genetic factors in DS phenotypes is supported by epidemiological studies of DS: specific cardiac defects were associated with smoking mothers (AVSD, TOF) [17], [18], folate pathways and folate supplementation have been proposed to interfere with the incidence of AVSD [19], [20], and an association between DS and CHD with global hypomethylation status has been found in a Dutch population-based case-control study [21].

We hypothesized that if the cardiac phenotypes observed in DS are caused, at least in part, by trisomy-induced gene dosage effects, it should be possible to genetically differentiate individuals with DS by their specific heart defects, as well as distinguishing those with heart defects from those without. Further, such genetic differences may be manifested in peripheral tissues: dermatoglyphic indicators used to characterize individuals with DS have been used to correctly classify them according to their heart status [22].

Several studies have suggested that LCLs can be used to detect biologically plausible correlations between candidate genes and various genetically-induced diseases. We collected blood samples from individuals with DS and controls (2N) and established an LCL biobank. From this cell bank, we selected LCLs from the following five types of donors: euploid individuals, individuals with DS and without CHD, individuals with DS and AVSD, individuals with DS and ASD, and individuals with DS and VSD. Microarrays and quantitative PCR were used to identify differentially expressed (DE) genes. Interestingly, principal component analysis was able to distinguish between cells from individuals with AVSD and those with ASD or VSD (combined group, hereafter ASD+VSD). Differential expression analyses allowed us to identify genes and pathways specifically dysregulated in AVSD and/or ASD+VSD.

## Results

### Cell lines and phenotypes of each group

Blood samples were collected at five different clinical centers to establish a Biobank from 190 controls and 550 individuals with DS. Recorded phenotypes included cardiac defects (AVSD, ASD, VSD, TOF, PDA, mitral valve prolapsus) and murmurs. To construct the CHD^−^ group individuals without murmurs were selected. For the CHD^+^ group individuals with only one heart malformation were selected. For the expression study, samples were selected from caucasian individuals with DS with the same age distribution (23.05±2.4) for CHD**^+^** and (24.66±2.2) for CHD**^−^**. These groups were also created with the same gender ratio (M/F = 1.1 for CHD**^+^**; M/F = 1.2 for CHD**^−^**). The same criteria (ethnicity, age, gender) were used to select a group of 2N controls.

### Differential expression between AVSD and ASD +VSD

**Figure 1:**
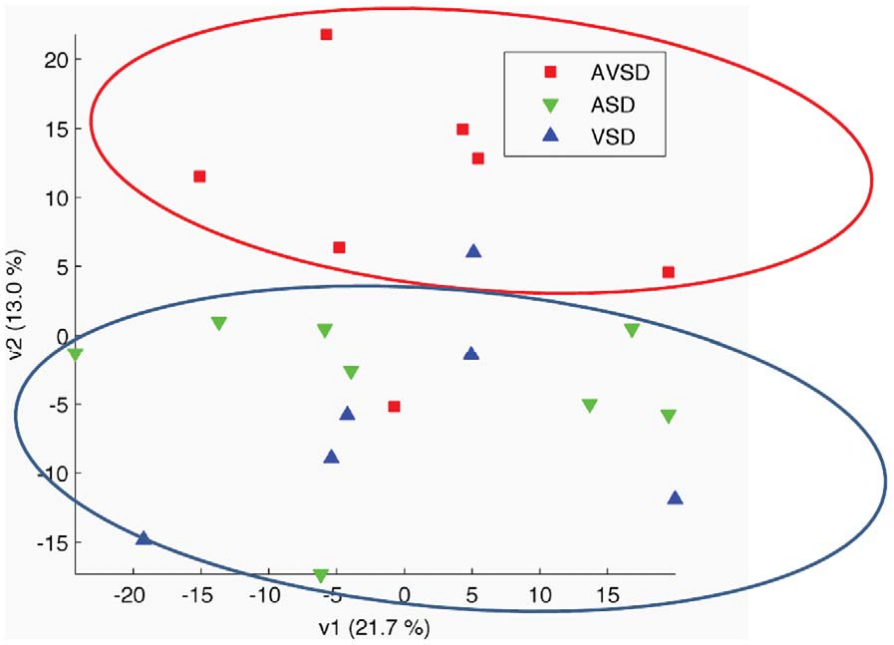
Principal component analysis of gene expression profiles of LCLs from individuals with DS and with CHD: red- AVSD (n = 7); green- ASD (n = 8); blue- VSD (n = 6). Red circle indicates AVSD, blue circle indicates ASD and VSD.

**Figure 2:**
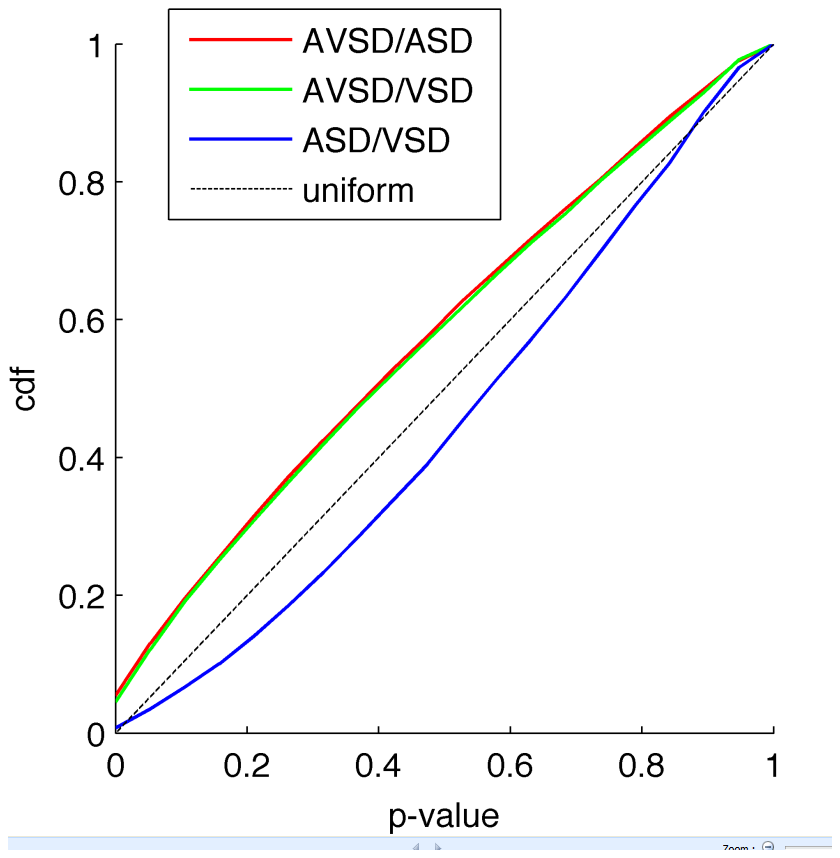
Classification of heart defects (CHD+) according to the distribution frequency of their p-values. Empirical cumulative distributions of p-values for the 2-by-2 Student tests A: AVSD/ASD; B: AVSD/VSD; C: ASD/VSD. Comparisons were obtained for 11 224 tests. The comparison to the uniform distribution shows that there is no overall differential expression between ASD and VSD (less small p-values than for an uniform distribution). Cdf: cumulative distribution function.

Using human pangenomic Illumina microarrays containing 48,701 probes, we obtained expression profiles from 43 samples: 21 from individuals with DS and with a heart defect (CHD**^+^**: 7 AVSD, 8 ASD, 6 VSD) and 22 with DS and without heart defect (CHD**^−^**). Among the 48,701 probes on the microarrays, 11,224 probes corresponding to 9,758 genes displayed significant expression in LCLs. Results from three independent experiments (43 total samples from people with DS) were analyzed to identify DE genes. Data were normalized using quantile normalization. A first comparison of gene expression profiles using principal component analysis (PCA) could not differentiate between the CHD**^+^** and the CHD**^−^** groups. We thus performed a PCA on the CHD**^+^** group only. This analysis showed that individuals with DS and AVSD could be separated from a second group corresponding to individuals with DS and ASD or VSD (Figure 1). However, PCA failed to separate between the ASD and VSD groups, even in the subspace of the first three principal components. A second analysis of the CHD**^+^** group consisted of two-by-two t-tests with a global estimate of the common variance, leading to 249 transcripts with p-value below 0.01 for the AVSD/ASD comparison, 192 transcripts for the AVSD/VSD comparison, and 33 for the ASD/VSD comparison. We performed a classification of heart defects (CHD+) according to the distribution frequency of their p-values. We calculated the empirical cumulative distributions of p-values for the 2-by-2 Student tests A: AVSD/ASD; B: AVSD/VSD; C: ASD/VSD. Comparisons were obtained for 11 224 tests. The comparison to the uniform distribution shows that there is no overall differential expression between ASD and VSD (less small p-values than for an uniform distribution).This analysis indicated that there were no significantly DE genes between ASD and VSD, and validated our choice to group ASD with VSD for further analysis (ASD+VSD group) (Figure 2).

Analysis of the data was then performed using ANalysis Of Variance (ANOVA) on the three following groups: the CHD**^−^** group, the AVSD group, and the ASD+VSD group. We considered genes with p<0.05 as DE, and, for the latter, we performed two-by-two comparisons with the CHD^−^ groups. This analysis revealed a group of DE genes in AVSD with respect to CHD**^−^** (889) and a group of DE genes in ASD+VSD with respect to CHD**^−^** (1016); the two groups exhibited a small overlap (119) (Table S1).

We also compared cells from 11 CHD**^−^** individuals with DS to cells from 12 controls (2N individuals) and found 1,766 DE genes with p<0.05. DS phenotypic variability may be related to dysregulation of genes with a causative effect (inducing the defect) or with a protective effect (compensating other negative effects). Detailed analysis of the two groups with heart defects (CHD**^+^** vs. CHD**^−^**) allowed us to group the DE genes into two classes. The first class (I) contained genes DE in CHD^+^ (AVSD or ASD+VSD) with a larger effect between CHD**^+^** and CHD**^−^** than between CHD^−^ and 2N; we further divided this class into two sub-classes (IA, IB) to differentiate genes with similar expression (ratio interval between 0.95 and 1.05) between CHD**^−^** and 2N individuals (i.e., genes that are not dysregulated in CHD**^−^**). The second class (II) contained genes DE in CHD**^+^** with expression levels closer to the those observed in 2N individuals than to those found in the CHD^−^ group; we also divided this into two sub-classes (IIA, IIB) to differentiate genes that presented similar expression (ratio interval between 0.95 and 1.05) between CHD**^+^** and controls (Table 1 and Table S2).

**Table 1:** Classification of differentially expressed genes according to heart phenotype and relative expression in Ts21 compared to 2N.

CHD^+^ categories	# DE genes:	group I genes:		group II genes:		Clusters on HSA21
		|CHD+−CHD−|>|CHD−−2N|		|CHD+−CHD−|<|CHD−−2N|		
		A	B	A	B	
	N probes/n genes	CHD^−^≠2N	CHD^−^ = 2N	CHD^+^≠2N	CHD^+^ = 2N	
	p<0.05	(p<0.05)	(p≥0.05)	(p<0.05)	(p≥0.05)	
**AVSD**	1006/889	295	336	64	0	PIGP-TTC3-DSCR3 (IA)
**ASD+VSD**	1136/1016	320	327	34	62	IFNAR1-GART-DONSON (IIA)

Group IA: |CHD**^+^**−CHD**^−^**|>|CHD**^−^**−2N| and CHD^−^≠2N; Group IB : |CHD^+^−CHD^−^|>|CHD^−^−2N| and CHD**^−^** = 2N; Group IIA: |CHD**^+^**−CHD**^−^**|<|CHD**^−^**−2N| and CHD**^+^**≠2N; Group IIB: |CHD**^+^**−CHD**^−^**|<|CHD**^−^**−2N| and CHD**^+^** = 2N.

### Validation by quantitative PCR

We then performed quantitative PCR on a set of LCLs including the ones analyzed in microarray experiments and LCLs from other individuals. Two reference genes were selected for these experiments: hypoxanthine phosphoribosyl transferase (HPRT) and polymerase (RNA) II (DNA directed) polypeptide A (POLR2A). Validation experiments were performed on genes showing high differential expression (ratio >1.3 or ratio <0.75), genes selected for their potential functional involvement in heart development, and genes from clusters on HSA21 (oligonucleotide sequences are listed in Table S3). We confirmed results obtained by microarray analysis both for genes with an increased expression in CHD^+^ and for genes with a decreased expression in CHD^+^ (R^2^ = 0.941, p<0.0001) (Table 2).

**Table 2:** Transcriptome signature of heart defects.

	Transcriptome signature of heart malformations		
genes	array ratios	array ratios	qPCR ratios
	CHD−/2N	AVSD/CHD−	AVSD/CHD−
AUTS2	0.86 (NS)	1.83*	1.73*(11/10), 1.55*(10/10), 1.76* (16/16),
			1.92*(18/18), 1.81*(18/18)
CNN2	0.9 (NS)	0.69*	0.82*(11/10)
DSCR3	1.21*	1.22*	1.27*(10/10), 1.19*(9/10)
DYNLT3	1.01(NS)	1.42*	1.68*(18/19), 1.71*(17/18)
NQO1	1.57*	0.62*	0.63*(20/19)
OFD1	0.99(NS)	1.39*	1.17 (NS)
PDIA4	1.14(NS)	0.74*	0.7*(11/10), 0.76*(17/17), 0.66*(20/20)
PIGP	1.31*	1.26*	1.35*(10/10), 2.2*(8/9), 1.22*(19/17),
			1.31*(17/18)
TTC3	1.35*	1.24*	1.39(12/11), 1.35(10/19), 1.34*(11/12),
			1.14*(18/20)
TUBB2B	1.01(NS)	1.79*	1.64*(18/18), 1.8*(18/18)

	CHD−/2N	ASD+VSD/CHD−	ASD+VSD/CHD−
ACTG1	0.96(NS)	0.63*	0.73*(11/11)
ALDOC	0.87(NS)	1.36*	1.56*(10/10), 1.56*(19/17), 1.54*(19/20)
CACYBP	1.07(NS)	0.73*	0.75*(11/10)
DTYMK	1.00(NS)	0.7*	0.76*(9/9), 0.74*(10/10), 0.8*(11/11)
ENO2	0.89(NS)	1.54*	1.34(NS)
GART	1.39*	0.83*	0.72*(11/11)
IFNAR1	1.21*	0.87*	0.78*(10/10), 0.58* (10/10)
PLTP	1.01(NS)	1.37*	1.32*(10/10)
RPL10A	0.84*	0.64*	0.71*(10/11)
TNFAIP2	1.02(NS)	1.49*	1.5*(9/9)

Comparison of ratios for DE genes up- or down-regulated in the AVSD or ASD+VSD group between (1) array ratio in the Ts21 CHD^−^ vs 2N, (2) array ratio in CHD^+^ vs CHD^−^, (3) q-PCR ratio from different experiments in CHD^+^ vs CHD^−^ using additional samples (total number in parentheses). Gene nomenclature, *AUTS2*: autism susceptibility candidate 2; *CNN2*: calponin 2; *DSCR3*: Down syndrome critical region gene 3; *DYNLT3*: dynein, light chain, Tctex-type 3; *NQO1*: NAD(P)H dehydrogenase, quinone 1; *OFD1*: oral-facial-digital syndrome 1; *PDIA4*: protein disulfide isomerase family A, member 4; *PIGP*: phosphatidylinositol glycan anchor biosynthesis, class P; *TTC3*: tetratricopeptide repeat domain 3; *TUBB2B*: tubulin, beta 2B; *ACTG1*: actin, gamma 1; *ALDOC*: aldolase C, fructose-bisphosphate; *CACYBP*: calcyclin binding protein; *DTYMK*: deoxythymidylate kinase (thymidylate kinase); *ENO2*: enolase 2 (gamma, neuronal); *GART*: phosphoribosylglycinamide formyltransferase, phosphoribosylglycinamide synthetase, phosphoribosylaminoimidazole synthetase; *IFNAR1*: interferon (alpha, beta, and omega) receptor 1; *PLTP*: phospholipid transfer protein; *RPL10A*: ribosomal protein L10a; *TNFAIP2*: tumor necrosis factor, alpha-induced protein 2.

### Chromosomal localization

We then analyzed the chromosomal localization of DE genes. Interestingly, genes DE in AVSD as compared to CHD^−^ preferentially mapped to chromosomes 7, 11, 16, 19, and 20. In addition, we identified a cluster that contained three contiguous genes mapped to HSA21: *PIGP (DSCR5)*, *TTC3*, and *DSCR3*. A numerical simulation to compute the probability of finding a triplet on chromosome 21, assuming that gene expression and regulation are independent of localization, showed that the triplet found on HSA21 in the AVSD group was significant (p = 0.039) (Table S4). These three genes belonged to class IA (genes DE in AVSD as compared to CHD^−^ with a larger effect between AVSD and CHD**^−^** than between CHD**^−^** and 2N, see Table 1). When analyzing genes DE in ASD+VSD as compared to CHD**^−^**, we found an enrichment of genes from chromosomes 11, 19, and 22. We also found a cluster of genes from HSA21, including *IFNAR1*, *GART*, and *DONSON*, mapping close to each other. These genes were from class IIA (genes with expression levels closer to the ones observed in 2N individuals than to the ones found in the CHD**^−^** group, see Table 1). The triplet on chromosome 21 in the ASD+VSD group was not significant (Table S4).

### Expression pattern, GO category and pathways enrichment analysis

A list of genes potentially expressed in fetal heart was built from the human and mouse microarray datasets from GEO. Although this list is not exhaustive, it allowed us to characterize expression in the heart as compared to other tissues. We compared this list to our list of DE genes, and found that 65% of DE genes in AVSD and 63% of DE genes in ASD+VSD were expressed in the heart with a significant enrichment (p = 5×10^−3^ for AVSD and p = 0.01 for ASD+VSD).

Gorilla software was used to calculate the enrichment of GO categories in DE genes. Comparison of genes expressed in LCLs (background set) with genes DE in AVSD as compared to CHD^−^ (target set) showed an enrichment of two GO categories corresponding to genes involved in the regulation of metabolic processes and zinc ion binding. Similar analysis performed on DE genes in ASD+VSD (as compared to CHD^−^) indicated an enrichment of three GO categories: biosynthetic processes, demethylation, and regulation of transcription by glucose (Table 3).

**Table 3:** GO categories in the CDH^+^ DE genes.

Heart defects	GO Term	Description	P-value	Enrichment (N, B, n, b)
**DE [AVSD/CHD^−^]**	GO:0008270	zinc ion binding (F)	7.22E-04	1.30 (8259,986,810,126)
	GO:0030665	clathrin coated vesicle membrane (C)	7.56E-04	3.71 (8259,22,810,8)
**DE [ASD+VSD/CHD^−^]**	GO:0016043	cellular component organization (P)	3.14E-05	1.25 (8264,1736,888,234)
	GO:0071840	cellular component organization or biogenesis (P)	3.98E-05	1.25 (8264,1759,888,236)
	GO:0071822	protein complex subunit organization (P)	1.26E-04	1.56 (8264,393,888,66)
	GO:0006909	phagocytosis (P)	4.40E-04	3.92 (8264,19,888,8)
	GO:0043933	macromolecular complex subunit organization (P)	4.67E-04	1.42 (8264,551,888,84)
	GO:0071526	semaphorin-plexin signaling pathway (P)	6.06E-04	7.45 (8264,5,888,4)
	GO:0007411	axon guidance (P)	6.94E-04	1.90 (8264,132,888,27)
	GO:0051592	response to calcium ion (P)	9.90E-04	2.84 (8264,36,888,11)
	GO:0032774	RNA biosynthetic process (P)	9.97E-04	1.46 (8264,407,888,64)
	GO:0017154	semaphorin receptor activity (F)	1.33E-04	9.31 (8264,4,888,4)
	GO:0032991	macromolecular complex (-C)	2.97E-05	1.22 (8264,2220,888,290)
	GO:0043234	protein complex (-C)	3.26E-04	1.21 (8264,1789,888,233)
	GO:0005881	cytoplasmic microtubule (-C)	3.71E-04	3.64 (8264,23,888,9)
	GO:0005829	cytosol (-C)	8.17E-04	1.22 (8264,1521,888,199)
	GO:0005829	cytosol (-C)	4.43E-04	1.26 (8256,1388,804,170)

Analysis of the enrichment of GO categories for the genes DE in the comparison between AVSD or ASD+VSD set of genes and CHD^−^ set of genes compared to the genes expressed in LCLs. Analysis used GOrilla software with a p-value threshold at 10^−3^. N - is the total number of genes, B - is the total number of genes associated with a specific null, n - is the number of genes in the group with heart defect, b - is the number of genes in the intersection.

Since pathway analysis is highly dependent on the gene annotations present in databases, we established lists of genes for pathways potentially related to heart development (Table S5), specifically the Wnt, Hedgehog, Notch, and Jak-stat pathways. In addition, we created lists of genes involved in angiogenesis/cardiogenesis and in the epithelial-to-mesenchymal transition, i.e., genes expressed in the progenitors of the mitral and tricuspid valves and the membranous interventricular septum since they are known to arise by an epithelial-mesenchymal cell transformation (EMT) from embryonic endothelial cells [23],[24]. Among genes DE in ASD+VSD as compared to CHD**^−^**, we found a significant enrichment in genes belonging or connected to the Hedgehog pathway and to the Jak-Stat pathway (Table 4, in bold). Interestingly, we also found a highly significant enrichment in cilia genes known to be critical for organ laterality and heart development [25] (and listed from McClintock [26]) among DE genes in the ASD+VSD cases but also in the AVSD cases (Table 4).

**Table 4:** Pathways analysis of DE genes in the CHD^+^.

	LCLs	AVSD/CHD^−^		ASD+VSD/CHD^−^	
pathways	N	n	enrichment p value	n	enrichment p value
**notch**	82	6	0.615	11	0.163
**wnt**	92	10	0.277	12	0.204
**jak-stat**	68	7	0.259	12	**0.008**
**hedgehog**	37	1	0.912	8	**0.012**
**epithel-mesenchym**	31	2	0.673	4	0.23
**angiogenesis/cardiogenesis**	17	2	0.703	2	0.428
**ciliome**	1134	134	**0.00017**	148	**0.00027**

N is the number of genes from the pathway in genes expressed in LCLs. n is the number of DE genes from the pathway in the AVSD or ASD+VSD set of genes. p is the p value for the enrichment calculated with 9,758 genes expressed in LCLs, 889 DE genes in AVSD group, and 1,017 DE genes in ASD/VSD group.

## Discussion

Identification of candidate genes or pathways involved in the occurrence of congenital heart defects in DS has been extensively pursued, but has failed to provide a clear picture of the genes responsible for the developmental anomalies observed in individuals with DS. In the mouse, the construction of models carrying partial trisomies for chromosomal regions orthologous to HSA21 have suggested that (1) trisomy of genes from HSA21—or their orthologs—may induce heart defects, as shown in Tc1 [27] and Ts65Dn [28],[29] mice, and (2) specific genes or regions may contribute heavily to heart defects [30],[31] in the murine models. In human, attempts to identify candidate genes have compared the transcriptomes of hearts from fetuses with or without DS [32]–[34]. However, these studies were performed either without screening for heart defects or without differentiating between various heart defects for a small number of cases and did not lead to the identification of genes DE in individuals with DS with CHD as compared to individuals with DS without CHD.

### Peripheral tissues carry signatures of heart defects

Using peripheral tissues for analysis of genetic changes allows a study to assess a larger number of individuals. Recent studies describe the use of LCLs to characterize expression changes in various genetic diseases including including Rett syndrome [35], nonspecific X-linked mental retardation [36], bipolar disorder [37], autism and fragile X syndrome [38] ^,^ [39], imprinting diseases [40], and cardiomyopathy [41]. A recent study of individuals with mosaicism for trisomy 21 showed that congenital heart defects were significantly correlated with the percentage of trisomy in lymphocytes [42]. Here, performing microarray experiments on RNAs extracted from LCLs of individuals with DS with and without CHD, we have shown that it was possible to classify DS carriers of heart defects.

### Alteration of signaling pathways differs according to specific heart defects

Within the group with heart defects (CHD**^+^**), it was possible to differentiate between the transcriptome of cells from individuals with DS carrying AVSD and those carrying ASD or VSD. With a p-value below 0.05, 889 genes were DE between AVSD and CHD^−^ and 1,016 genes between ASD+VSD and CHD**^−^**. The two groups had a modest overlap of 10–15%, indicating that, for the most part, different molecular causes contribute to these morphologically-different cardiac malformations.

Genes that exhibit stronger dysregulation in those with heart defects are good candidates to explain the defects, particularly considering alteration of the pathways including or associated with these genes. However, expression analysis also revealed, at least in LCLs, the presence of genes (group II) with an expression level closer to the expression observed in controls than in CHD**^−^**: these results may suggest that over-dysregulation of these genes in CHD^−^ exerts a protective effect against the dysregulation of other genes directly involved in heart defects.

### Chromosomal localizations of dysregulated genes is not always random

Among DE genes located on HSA21, two clusters were identified: the AVSD cluster (*PIGP-TTC3-DSCR3*) and the ASD+VSD cluster (*IFNAR1-GART-DONSON*). These genes are included in the narrowed candidate region containing potentially relevant genes for heart defects in the trisomy 16 mouse models [31]. Moreover, SNP-to-expression associations have been found in *cis* for TTC3 and PIGP(DSCR5) [43]. Screening the chromosomal location of DE genes revealed an overrepresentation of genes located on specific chromosomes, specifically chromosomes 7, 11, 16, 19, and 20 for the AVSD group and 11, 19, and 22 for the ASD+VSD group. The presence of these contiguous clusters of genes points to possible common regulatory processes involved in CHD likely arising from local chromatin modifications.

### Dysregulated genes or pathways are associated with cardiogenesis and/or cardiac defects

Among DE genes that can be considered as candidate genes there is a gene encoding an ubiquitin E3 ligase (*TTC3*). Interestingly, E3 ligases have been implicated in the etiology of human cardiovascular diseases: *FBXO25* targets cardiac transcription factors, indicating that cardiac protein homeostasis has a pivotal impact on cardiac development that is dependent on the ubiquitin-proteasome system [44]; increased levels of *TTC3* may interact with similar processes.

There also exist potential synergistic effects. For example, co-regulation of *TTC3* and *AUTS2* has been shown during the differentiation of human embryonic stem cells (hESC) into cardiomyocytes. *TTC3* and *AUTS2* are upregulated during the first step of differentiation of hESC-derived cardiomyocytes into beating embryoid bodies that highly express cardiac transcriptional regulator genes [45]. Interestingly, *TTC3* and *AUTS2*, which appear to be involved in common mechanisms, are both DE in cells from individuals with DS with AVSD.

GO analyses of differential expression in AVSD revealed an enrichment for genes linked to the formation of clathrin-coated vesicle membranes; clathrin-mediated membrane trafficking is essential for cell organisation and likely to be involved in various steps of heart formation. Additionally, we noted an enrichment in genes from the semaphorin-plexin signaling pathway for ASD+VSD: knockdown of semaphorin3D reduces the size of the primary heart field [46], and semaphorin3C appears to be regulated by *Tbx1*, *Pitx2*, and *Gata6*—critical regulators of secondary heart field development [47]; PlexinA1-SemaD6 interaction are involved in myocardial organization and growth [48].

Enrichment of DE genes in pathways that are known to be involved in the differentiation of the heart were analyzed more specifically than with the GO approaches. We developed lists of genes in involved pathways by compiling three different sources (AMIGO, KEGG, and SAB). We identified an enrichment in the Hedgehog pathway only in the ASD+VSD group. Hedgehog, and particularly the Sonic Hedgehog (Shh) pathway, is proposed to be involved in heart development. Many processes (proliferation/differentiation, migration, transition) and cell types participate in heart development, from the heart tube to the four-chamber heart. Of particular interest are the secondary heart field and neural crest [49],[50],[51], [52]. The secondary heart field, composed of different sub-domains, is the target of multiple signals that emerge from outside the cardiac progenitors. Among these signals, alteration in the level of Shh secreted by the pulmonary endoderm may affect the development of the atrial septum [53] ^,^ [54]. Interestingly, abnormal SHH expression has been observed in fetuses with DS presenting an increased nuchal translucency [55], a feature frequently associated with heart defects. Moreover, altered response to Shh is suggested to be associated to a deficit in neural crest development in Ts65Dn mice [56]. Thus, Shh signaling may contribute, via various cells, in the ASD or VSD anomalies observed in DS.

The Jak-stat pathway was also found to be dysregulated in the ASD+VSD group. A recent study using *Drosophila* as a model system found that Jak-stat controls the expression of *tin*, a member of the NK2 transcription factor family, and modulates heart precursor diversification [57]. Further, a study of proteins secreted by hESCs identified a potential role of the Jak-stat pathway in cardiogenesis [58]. Therefore, even subtle alterations in this pathway could modify very early heart development.

The primary cilium has recently been shown to play a crucial role in vertebrate development, including that of the heart [25],[59],[60]. Cilia also appear to be involved in Hedgehog signaling. Interestingly, although human mutations in cilia genes were first implicated in LR asymmetry inducing heterotaxy, they may also lead to heart defects including AVSD, ASD, or VSD, as in Ellis-van Creveld syndrome [61]. We therefore tested whether cilia genes were DE in cells from CHD**^+^** individuals as compared to CHD^−^. Using the database constructed from mouse cilia genes [26], we found an enrichment in cilia genes for the two CHD**^+^** groups (AVSD and ASD+VSD), with an overlap of only 16 genes (12%) between the two sets. Interestingly, *TUBB2B* and *OFD1* (Table 2), as well as the HSA21 gene *TSGPA2/RSPH1* (or meichroacidin [62]) are upregulated in cells from AVSD cases.

Together, the results of this study highlight genes already known to participate in heart defects, suggest new involvement of specific pathways in heart morphogenesis, and open new perspectives to understanding cardiogenic alterations present in individuals with Down syndrome. Further studies on embryonic samples at different stages of development will be necessary to identify the mechanisms at the origin of these different cardiac defects.

## Materials and Methods

### Individuals and cell lines

LCLs were derived from peripheral blood lymphocytes collected from individuals with DS by Genethon platform. Parents of individuals with DS gave written informed consent, and the French biomedical ethics committees gave their approval for this study (Comité de Protection des Personnes dans la Recherche Biomédicale number 03025 and CPP SDE I agreement N° AC-2009-12). Heart phenotypes were established from the medical records either on surgery or echocardiography. Cell lines from control individuals were also obtained, with written informed consent, for comparison of chromosome 21 gene expression profiles. Immortalization was performed in Cell department of Genethon with the same EBV batch. All cell lines were karyotyped to confirm their trisomic or euploid status and to verify that immortalization by the Epstein-Barr virus (EBV) did not produce any additional chromosomal rearrangement beyond trisomy 21. Lymphoblastoid cells were grown in Opti-MEM supplemented with 5% fetal calf serum (Life Technologies) and antibiotics (Penicilline, Streptomycine).

### RNA extraction

Frozen vials from previously immortalized cell lines were cultured in 5% fetal calf serum in a 5% CO_2_ incubator. Cells were harvested at approximately 60% confluency by centrifugation to a cell pellet of on average 10^7^ cells. The cell pellet was resuspended in lysis buffer and cell aggregates were dissociated by careful pipeting, and the Macherey-Nagel mini kit was used to extract total RNA. RNA was quantified using a NanoDrop and checked for quality using the Agilent Bioanalyser. Individual samples were randomised before RNA extraction, amplification, and hybridisation to Illumina Human WG-6 Sentrix BeadArray arrays. Arrays were hybridised with labelled cRNA material and scanned according to manufacturer's instructions.

### Expression data

Expression data were obtained using Illumina Human-6 v2 expression beadchips with 47,701 probes (Illumina Inc., San Diego, CA) on cRNAs from 21 individuals with heart defects (CHD**^+^**: 7 AVSD, 8 ASD, 6 VSD) and 22 without heart defects (CHD**^−^**). In a second experiment, Illumina Human HT-12 v3 beadchips with 48,803 probes were used to compare 11 individuals with DS without heart defects (CHD**^−^**) to 12 controls (2N individuals). For Illumina BeadArray assay, cRNA (500 ng) was synthesized with the Illumina RNA Amplification Kit (Ambion, Austin, TX, USA), purified, labeled, and hybridised according to the manufacturer's instructions. Then, arrays were scanned using the Illumina BeadArray reader (500GX, Illumina), and scanned images were imported in the BeadStudio software version 3.1.3.0 (Illumina). For the expression array, unprocessed data were collected from BeadStudio and processed using variance-stabilizing transformation (VST) method and quantile normalization with the R bead array package. Microarray data have been deposited in GEO database with the number GSE34459.

### Pathways analysis

Gene content of pathways were defined according to three databases: AMIGO, KEGG, and SAB. Gene lists were constructed by including aliases (GeneCards V3 database, http://www.genecards.org/).

### Real-time quantitative Reverse Transcription PCR

qRT-PCR was used to confirm the results obtained by expression arrays. The number of samples was increased in comparison with microarrays to increase the statistical power of the analysis. cDNAs were synthesized using 1 µg of total RNA with reverse transcriptase enzyme (Takara, Ozyme) in combination with oligo-dT and random hexamer priming, and qPCR was performed as described elsewhere (Roche 480). Primer details are in Table S3. Expression of the genes of interest was normalized by using the program. Two normalization genes were selected within this program: *HPRT* and *POLR2A*. Relative quantification was performed using standard curves, followed by adjustment with a normalization factor (REST 2009 v2.0.13). Average relative expression of genes of interest in studied groups was compared to controls (either 2N or CHD^−^).

### Statistical analysis

Because they were obtained on two different versions of the Illumina beadarrays, the expression data sets were analyzed separately. Both were normalized using quantile normalization, and expression levels were considered significant when their p-values were below 1%. Principal component analyses (PCA) were performed on the transcripts whose expression levels were significant.

In the data set of individuals with DS only, we first analyzed the subset of 21 individuals with heart defects (CHD**^+^**: 7 AVSD, 8 ASD, 6 VSD) with ANOVA, followed by two-by-two t-tests with a global estimate of the common variance, on the 11,224 transcripts showing a significant expression level across at least 4 arrays in each of the three groups. For the ASD/VSD comparison, only 33 transcripts were found differentially expressed (DE), i.e., far less than expected under the global null hypothesis (112); therefore, the ASD and VSD group were merged into one, the ASD+VSD group. Then, a second ANOVA was conducted on the totality of the first data (22 CHD^−^, 7 AVSD, and 14 ASD+VSD) again followed by two-by-two t-tests.

The data set with 11 CHD^−^ individuals with DS and 12 controls was analyzed with a t-test on the 14,649 transcripts showing significant expression across at least 9 arrays in each group.

## Supporting Information

Table S1 Differentially expressed genes in LCLs from DS with and without heart defect.
(XLS)

Table S2 Classification of differentially expressed genes according to their relative level of expression in CHD+ as compared to CHD.
(XLS)

Table S3 Oligonucleotide sequences for qRTPCR. Gene nomenclature.
(XLS)

Table S4 Genomic localization of DE genes: Enrichment in DE genes from specific chromosomes and probability to find contiguous genes as doublets or triplets.
(XLS)

Table S5 List of genes involved in pathways associated with heart morphogenesis.
(XLS)
